# Anti-Atherosclerotic Effects of Fruits of *Vitex rotundifolia* and Their Isolated Compounds via Inhibition of Human LDL and HDL Oxidation

**DOI:** 10.3390/biom9110727

**Published:** 2019-11-12

**Authors:** Jae-Yong Kim, Sang Hee Shim

**Affiliations:** College of Pharmacy, Duksung Women’s University, Seoul 01369, Korea; kjaey0331@naver.com

**Keywords:** *Vitex rotundifolia* fruits, casticin, luteolin, low-density lipoprotein, high-density lipoprotein, oxidation, atherosclerosis

## Abstract

Low-density lipoprotein (LDL) and high-density lipoprotein (HDL) oxidation are well known to increase the risk for atherosclerosis. In our ongoing research on natural products with inhibitory activities against oxidation of lipoproteins, fruits of *Vitex rotundifolia* were found to be highly active. There is no report on the effects on LDL and HDL oxidation. Herein, we investigated the inhibitory effects of *V. rotundifolia* fruit extract and its six compounds, which are: (**1**) artemetin, (**2**) casticin, (**3**) hesperidin, (**4**) luteolin, (**5**) vitexin, and (**6**) vanillic acid, against LDL and HDL oxidation. The LDL and HDL oxidations were determined by measuring production of conjugated dienes and thiobarbituric acid reactive substances, amount of hyperchromicity and carbonyl content, change in electrical charge, and apoA-I aggregation. In addition, the contents of the compounds in the extracts were analyzed using HPLC-DAD. Consequently, extracts of *Vitex rotundifolia* fruits and compounds **2** and **4** suppressed oxidation of LDL and HDL, showing inhibition of lipid peroxidation, decrease of negative charges in lipoproteins, reduction of hyperchromicity, decrease in carbonyl contents, and prevention of apoA-I aggregation. In particular, compounds **2** and **4** exhibited more potent inhibitory effect on oxidation of LDL and HDL than the extracts, suggesting their protective role against atherosclerosis via inhibition of LDL and HDL oxidation. The contents of artemetin, casticin, and vanillic acid in the extracts were 1.838 ± 0.007, 8.629 ± 0.078, and 1.717 ± 0.006 mg/g, respectively.

## 1. Introduction

Atherosclerosis is the narrowing and stiffening of the arteries, caused by the accumulation of cholesterol, fatty substances, calcium, and other substances in the inner walls of the arteries, which is the usual cause of heart attack, heart failure, cardiac ischemia, stroke, and peripheral vascular disease, and is the leading cause of morbidities and mortalities worldwide [1,2]. Many studies have been conducted to investigate the relationship between incidence of atherosclerosis and lipoproteins [3,4]. Low-density lipoprotein (LDL) and high-density lipoprotein (HDL) are two major lipoproteins in human plasma. LDL is the main blood carrier of cholesterol for delivery to peripheral tissues, while HDL mediates the reverse cholesterol transport, which is the process of cholesterol movement from tissues back to the liver, and these transport processes are necessary for efficient homeostasis of cholesterol in the human body [5]. However, the levels of LDL or HDL and their modification act as an atherosclerotic risk factor, and are associated with an increased incidence of atherosclerosis.

Many studies have reported that the elevation of serum LDL level and its oxidation are strongly related with an increased risk of developing atherosclerosis [6,7]. In particular, oxidized LDL (Ox-LDL) is known to play an important key role in the initiation and progression of atherosclerosis, and is also well established as a useful marker for cardiovascular disease. [6]. Ox-LDL increases proliferation of vascular smooth muscle cells (VSMCs) and promotes adherence of monocytes to the endothelium [8]. In RAW264.7 mouse macrophages, oxLDL increased reactive oxygen species (ROS) production and elevation of lectin-like oxidized low-density lipoprotein receptor-1 (LOX-1) [9]. In addition, ox-LDL causes cytotoxicity for all cells involved in atherosclerosis such as lymphocytes, monocyte-derived macrophages, endothelial cells, and smooth muscle cells [10,11,12]. Besides, the elevation of oxLDL levels in plasma are well known to be associated with the incidence of acute myocardial infarction (AMI), coronary heart disease (CHD), metabolic syndrome, diabetes mellitus, chronic congestive heart failure, and hypertension [13,14,15].

On the other hand, high levels of HDL have a protective effect on atherosclerosis. The Framingham heart study, a long-term cardiovascular cohort study, found that increased levels of HDL-C were powerfully and independently related to inhibition of risk for CHD [16,17]. In addition, many previous studies have reported that HDL plays a major role in reducing the risk of atherosclerosis via antioxidative, reverse cholesterol transport, anti-inflammatory, antithrombotic properties, anti-LDL oxidation, and endothelial cell maintenance functions [18,19,20,21]. However, HDL can easily be modified and impaired through a variety of factors, including oxidation, which causes the risk of atherosclerosis through increased cytotoxicity and loss of antiatherogenic activities [22,23]. Furthermore, modified HDLs have been reported to increase in patients with a variety of diseases, including atrial fibrillation, rheumatoid arthritis, and myocardial infarction [24,25,26]. Therefore, the discovery of LDL and HDL oxidation inhibitors could be a good strategy for the prevention of heart diseases.

*Vitex rotundifolia*, belonging to the Verbenaceae family, is a branched, sprawling shrub that grows on beaches, rocky shorelines, and sand dunes and is widely distributed in Korea, India, China, and Japan [27]. The fruit of *V. rotundifolia,* called “*Man HyungJa”*, is well known as a folk medicine for the treatment of cold, headache, migraine, eye pain, neuralgia, and premenstrual syndrome in Korea and China [28]. The fruits of *V. rotundifolia* have been known to contain diverse constituents such as iridoids, phenylpropanoids, flavonoids, lignans, and diterpenes [29]. Among them, flavonoids are well known compounds that present a multitude of biological and pharmacological activities such as antioxidant, anti-inflammatory, antiosteoporosis, anticancer, and antiviral effects [30,31,32,33]. In particular, casticin, a major flavonoid from *V. rotundifolia*, has been reported to have various biological and pharmacological activities including antitumor, neuroprotective, anti-inflammatory, and analgesic activities [34]. Although *V. rotundifolia* extract and its flavonoids have various pharmacological roles in the human body, their antiatherosclerosis function through LDL and HDL oxidation have not been elucidated yet. Herein, we demonstrate the inhibitory effects of ethanol extract of *V. rotundifolia* and its flavonoids on oxidation of LDL and HDL. Furthermore, we investigate the amounts of the active compounds in the extracts of *V. rotundifolia*.

## 2. Materials and Methods 

### 2.1. Plant Materials

In October 2018, the dried *V. rotundifolia* fruits were purchased at Kyungdong Oriental Market (Seoul, South Korea) and botanically identified by the corresponding authors. A voucher was deposited at the pharmacognosy laboratory of College of Pharmacy, Duksung Women’s University (specimen No. NPC 6-5). An amount of 30 g of dried *V. rotundifolia* fruits was extracted three times with 1 L of MeOH during 1 h at 50 °C and the solvents were evaporated in vacuo at 40 °C, yielding the MeOH extract (1.5 g).

### 2.2. Standards and Reagents 

The standard compounds, artemetin (**1**), casticin (**2**), hesperidin (**3**), luteolin (**4**), vitexin (**5**), and vanillic acid (**6**), were isolated from the extract of *V. rotundifolia* fruits by emeritus professor Sam Sik Kang from Seoul National University. The compounds were identified by NMR and their purities were assessed by NMR and TLC before use for this study. Trichloroacetic acid (TCA), 1,1,3,3-tetramethoxypropane, copper sulfate (CuSO_4_), bovine serum albumin (BSA), and dialysis tubing cellulose membrane were purchased from Sigma Aldrich (St. Louis, MO, USA). Coomassie brilliant blue R-250 and guanidine hydrochloride were obtained from Tokyo Chemical Industry (Tokyo, Japan). 2,4-Dinitrophenylhydrazine (DNPH) was purchased from Junsei Chemical Co., Ltd (Tokyo, Japan). Acetic acid and sodium chloride were purchased from Merck (Darmsstadt, Germany).

### 2.3. Isolation of LDL and HDL from Human Plasma

Human whole blood samples were acquired from young and healthy male volunteers and approved by the Institutional Review Board (IRB) at Duksung Women’s University (IRB No. 2018-007-001). Blood was collected using the Vacutainer (BD sciences, Franklin Lakes, NJ, USA) containing ethylenediaminetetraacetic acid (EDTA, final concentration 1 mM) for each individual. Plasma was separated using high-speed centrifugation for 10 min at 4 °C at 1690× *g* (5810R; Eppendorf, Hamburg, Germany). Native low-density lipoprotein (LDL; *d* = 1.019–1.063 g/mL) and high-density lipoprotein (HDL; *d* = 1.125–1.225 g/mL) were isolated from the plasma by sequential ultracentrifugation in accordance with standard protocols [35]. Briefly, plasma was centrifuged for 22 h at 10 °C at 235,000× *g* using an LE-80 (Beckman, CA, USA) at the Instrumental Analysis Center of Duksung Women’s University. The LDL and HDL were extensively dialyzed three times for 24 h at 4 °C against Tris buffer containing 140 mM NaCl, 10 mM Tris–HCl, and 5 mM EDTA (pH 7.4). The protein concentration of LDL and HDL were determined according to the Lowry assay with slight modification [36].

### 2.4. Measurement of the Formed Conjugated Dienes (CD)

Formation of LDL and HDL conjugated dienes induced by copper ions was continuously monitored by measuring the absorbance at 234 nm at 37 °C using a Spectra Max 190 spectrophotometer (Molecular Devices, CA, USA) [37]. Native LDL (50 μg of protein/mL) and HDL (200 of μg protein/mL) were incubated with CuSO_4_ (at 10 μM) separately, under the presence of *V. rotundifolia* fruits extract (at 10 and 100 μg/mL, respectively), six isolated compounds (at 40 μM), or compound **2** and **4** (at 10 and 20 μM, respectively) in a medium containing 10 mM phosphate buffer (pH 7.4).

### 2.5. Measurement of Thiobarbituric Acid Reactive Substances (TBARS)

Native LDL and HDL (500 μg of protein/mL each) with CuSO_4_ (at 10 μM) in the presence of *V. rotundifolia* fruits extract (at 10 and 100 μg/mL, respectively), six isolated compounds (at 40 μM), or compound **2** and **4** (at 10 and 20 μM, respectively) were incubated for 4 h at 37 °C. After oxidation, 0.5 mM EDTA (pH 8.0) was added to terminate the reaction with nitrogen gas. Afterward, trichloroacetic acid (TCA) solution (20%) was added to HDL samples and reaction mixtures were incubated with thiobarbituric acid (TBA) solution (0.67% TBA in 0.05 N NaOH). The mixture was heated in a water bath at 90 °C for 20 min. The mixed samples were cooled at 4 °C and centrifuged at 848 *g* for 15 min. After centrifugation, the supernatant was measured at 532 nm using a UV-visible spectrophotometer Spectra Max 190 (Molecular Devices, CA, USA).

### 2.6. Measurement of Relative Electrophoretic Mobility (REM)

Electrophoretic mobilities of oxidized LDL and HDL were determined using 0.5% agarose gel. The electrophoresis was performed at 100 V for 40 min in a TAE buffer (40 mM Tris-Acetate, 1 mM EDTA, pH 8.0), after which the gels were fixed using a fixative solution (ethanol:acetic acid:distilled water, 60:10:30, *v*/*v*/*v*) for 30 min to 2 h. Afterward, the gels were dried in an oven at 80 °C for 1 h, followed by staining with 0.15% Coomassie Brilliant Blue R-250 to visualize the LDL and HDL band [38].

### 2.7. Measurement of apoA-I Aggregation

After oxidation, each HDL sample was denatured with Laemmli sample buffer and 2-mercaptoethanol (15:1, *v*/*v*) at 90 °C for 5 min. ApoA-I aggregation was performed by 12% SDS-PAGE. Afterward, the gels were stained with 0.15% Coomassie Brilliant Blue R-250 to visualize apoA-I in HDL [39].

### 2.8. Measurement of UV Absorbance

UV-visible spectrophotometer Spectra Max 190 (Molecular Devices, CA, USA) was used to measure UV absorbance of native or oxidized LDL and HDL at 280 nm. According to the following equation, hyperchromicity at 280 nm was calculated: % Hyperchromicity at 280 nm = [(Absorbance of oxidized sample − Absorbance of native or compound-treated sample)/Absorbance of oxidized sample] × 100 [40].

### 2.9. Measurement of Protein-Bound Carbonyl Groups

Protein-bound carbonyls were determined spectrophotometrically with the use of the carbonyl-specific reagent DNPH [41,42]. Amounts of 250 μL of native LDL or HDL and each oxidized lipoprotein were mixed with 350 μL of 7 mM DNPH in 2 N HCl. After 1 h at room temperature for formation of DNP-hydrazones, 600 μL of trichloroacetic acid (20% *w*/*v*) was added. The mixture was centrifuged at 9425× *g* for 10 min to obtain the pellet. The pellet was washed three times with 1 mL of ethanol/ethyl acetate (1:1. *v*/*v*) in order to remove unreacted DNPH. Afterward, the pellet was dissolved in 600 μL of a 6 M guanidine hydrochloride solution in 20 mM phosphate buffer. The DNPH samples were read at 379 nm using a UV-visible Spectra Max 190 spectrophotometer (Molecular Devices, CA, USA) and carbonyl concentration of each sample was calculated using a ε379 nm = 22,000 M^−1^∙cm^−1^ [43].

### 2.10. Sample Preparation for HPLC Analysis

All sample solutions were dissolved in methanol. Standard stock solutions of the compounds were prepared in a concentration of 1 mg/mL. Subsequently, a calibration curve of the standard solution at various concentration levels of 7.8125–500 μg/mL for artemethin (**1**), castin (**2**), and vanillic acid (**6**) were obtained by serial dilution with methanol. The extract was prepared in a concentration of 10 mg/mL. All of the analytical solutions were filtered through 0.45 µm RC-membrane syringe filter (Sartorius, Germany).

### 2.11. Optimization of Chromatographic Conditions

The Agilent 1260 infinity HPLC system, which consisted of a G1311C quaternary pump, a G1329B auto sampler, a G1316A column oven, and a G1315D photodiode array (PDA) detector, and Agilent ChemStation software (Agilent Technologies, Santa Clara, CA, USA), was used for HPLC analysis. The chromatography was performed using a ZORBAX SB-C18 (250 mm × 21.2 mm I.D., 5 µm, Agilent Technologies) column at 30 °C and detected at 280 nm of UV wavelength. The mobile phase consisted of water containing 0.2% formic acid (solvent A) and acetonitrile (solvent B) with gradient elution of 0–20 min, 8% B; 20–25 min, 8%–25% B; 25–28 min, 25%–27% B; 28–35 min, 27%–37% B; 35–40 min, 37%–40% B; 40–52 min, 40% B; 52–60 min, 40%–60% B; 60–65 min, 65%–100% B on a flow rate of 0.8 mL/min. Before the injection of the next sample, the column was re-equilibrated with the initial gradient of solvents for 7 min.

### 2.12. Statistical Analyses

Data were presented as the mean ± standard deviation (SD). Statistical significance was determined using analysis of variance (ANOVA), followed by Bonferroni multiple testing correction. A P-value less than 0.05 was considered statistically significant.

## 3. Results

### 3.1. Inhibitory Activity of Conjugated Dienes and TBARS Formation by V. rotundifolia Fruit Extract and Its Compounds on LDL and HDL Oxidation

**Figure 1:**
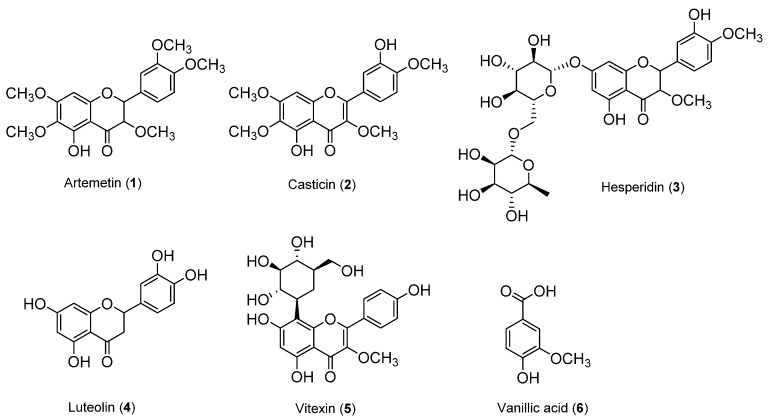
Chemical structures of compounds **1**–**6**; (**1**) artemetin, (**2**) casticin, (**3**) hesperidin, (**4**) luteolin, (**5)** vitexin, and (**6**) vanillic acid.

**Figure 2:**
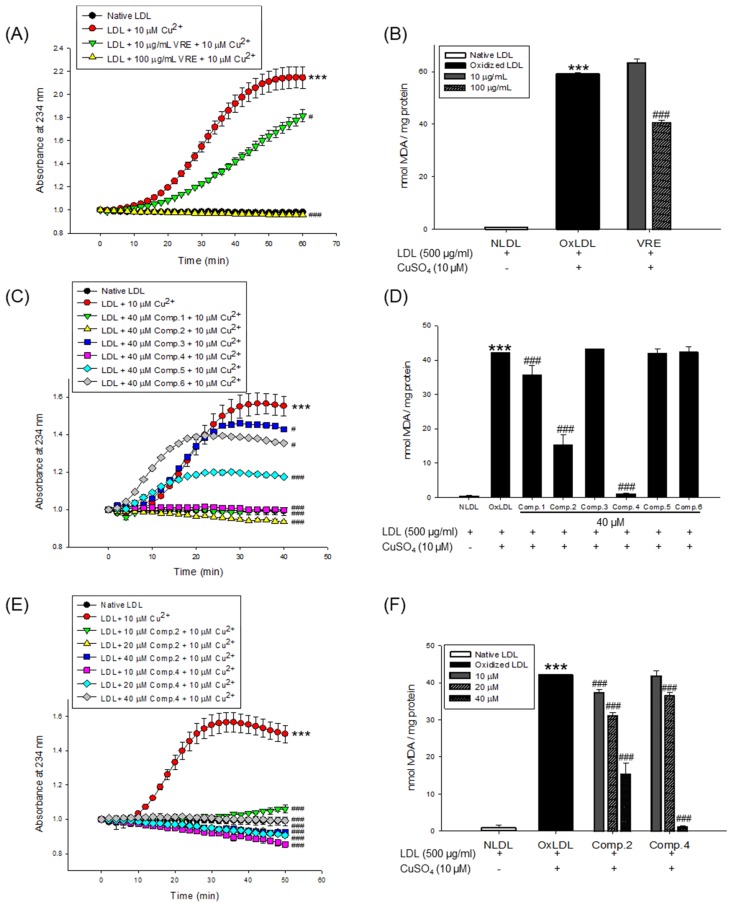
Effect of *V. rotundifolia* fruit extract and its six compounds on Cu^2+^-induced LDL oxidation. (**A**) Continuous monitoring of the conjugated diene levels by absorbance at 234 nm (A_234_) during 10 μM copper-ion-mediated LDL oxidation in the presence of *V. rotundifolia* fruit extract (at 10 and 100 μg/mL). (**B**) Effects of *V. rotundifolia* fruit extract (at 10 and 100 μg/mL) in TBARS production during LDL oxidation induced by CuSO_4_ for 4 h at 37 °C. (**C**) Conjugated diene levels by absorbance at 234 nm (A_234_) during 10 μM copper-ion-mediated LDL oxidation in the presence of six compounds (at 40 μM). (**D**) Effects of six compounds (at 40 μM) in TBARS production during LDL oxidation induced by CuSO_4_ for 4 h at 37 °C. (**E**) Conjugated diene levels by absorbance at 234 nm (A_234_) during 10 μM copper-ion-mediated LDL oxidation in the presence of low concentration compounds **2** and **4** (10, 20, and 40 μM). (**F**) Effects of low concentration compounds **2** and **4** (10, 20, and 40 μM) in TBARS production during LDL oxidation induced by CuSO_4_ for 4 h at 37 °C. These data are expressed as mean ± SD of three independent experiments. ***p < 0.001 vs. NLDL (native LDL); ^#^*p* < 0.05 vs. OxLDL (oxidized LDL); ^###^*p* < 0.001 vs. OxLDL.

**Figure 3:**
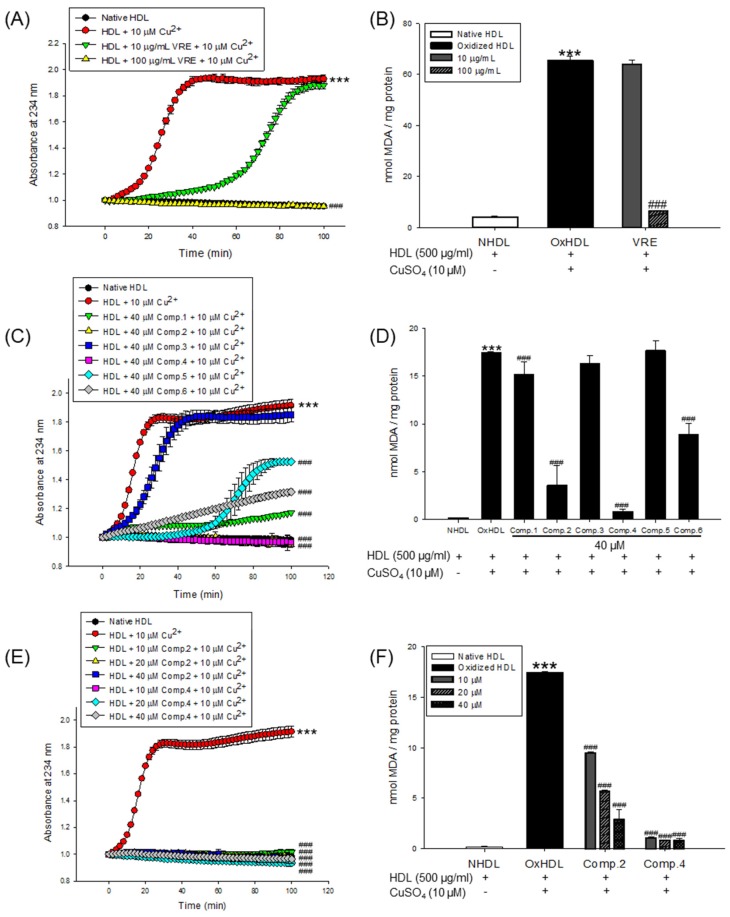
Effect of *V. rotundifolia* fruit extract and its six compounds on Cu^2+^-induced HDL oxidation. (**A**) Continuous monitoring of the conjugated diene levels by absorbance at 234 nm (A_234_) during 10 μM copper-ion-mediated HDL oxidation in the presence of *V. rotundifolia* fruit extract (at 10 and 100 μg/mL). (**B**) Effects of *V. rotundifolia* fruit extract (at 10 and 100 μg/mL) in TBARS production during HDL oxidation induced by CuSO_4_ for 4 h at 37 °C. (**C**) Conjugated diene levels by absorbance at 234 nm (A_234_) during 10 μM copper-ion-mediated HDL oxidation in the presence of six compounds (at 40 μM). (**D**) Effects of six compounds (at 40 μM) in TBARS production during HDL oxidation induced by CuSO_4_ for 4 h at 37 °C. (**E**) Conjugated diene levels by absorbance at 234 nm (A_234_) during 10 μM copper-ion-mediated HDL oxidation in the presence of low concentration compounds **2** and **4** (10, 20, and 40 μM). (**F**) Effects of low concentration compounds **2** and **4** (10, 20, and 40 μM) in TBARS production during HDL oxidation induced by CuSO_4_ for 4 h at 37 °C. These data are expressed as mean ± SD of three independent experiments. ***p < 0.001 vs. NHDL (native HDL); ^#^*p* < 0.05 vs. OxHDL (oxidized HDL);^###^*p* < 0.001 vs. OxHDL (oxidized HDL).

The inhibitory effects of the *V. rotundifolia* fruit extract and its six compounds (Figure 1) on LDL and HDL oxidation were assessed by measuring the total amounts of CD and TBARS produced as a result of the oxidation of LDL and HDL, which are the indicators of lipid peroxidation. Oxidized LDL and HDL induced by copper ion showed production of maximum CD formation and promoted lag phase compared to the native state (Figure 2A and Figure 3A, respectively). However, LDL treated with *V. rotundifolia* fruit extract (at 10 and 100 μg/mL) or its six compounds (at 40 μM) remarkably decreased the level of CD, and the extract or compounds (**1**, **2**, and **4**) further extended the lag time compared to oxidized LDL (Figure 2A,C,E). Similarly, HDL treated with the extract (at 10 and 100 μg/mL) and 40 μM compounds (**1**, **2**, and **4**–**6**) remarkably decreased CD formation and delayed lag time compared with oxidized HDL (Figure 3A,C,E). In addition, *V. rotundifolia* fruit extract (at 100 μg/mL) significantly inhibited the formation of MDA by copper-induced LDL oxidation (Figure 2B). The amount of 40 μM of compounds (**1**, **2**, and **4**) or compounds (**1**, **2**, **4**, and **6**) also suppressed MDA production on LDL and HDL oxidation (Figure 2D and Figure 3D, respectively). In particular, compounds **2** and **4** had a strong ability to inhibit CD and MDA formation on LDL and HDL oxidation by copper ions (Figure 2C,D and Figure 3C,D, respectively). Since compounds **2** and **4** showed strong activities, low concentrations of the compounds (10 and 20 μM) were treated to LDL and HDL. LDL with compound **2** (at 10 and 20 μM) and compound **4** (at 20 μM) and HDL with compounds **2** and **4** (at 10 and 20 μM) remarkably inhibited the generation of CD and TBARS (Figure 2E,F and Figure 3E,F).

### 3.2. Effects of V. rotundifolia Fruit Extract and Its Compounds on UV Absorption and Carbonyl Content of Oxidized LDL and HDL

**Figure 4:**
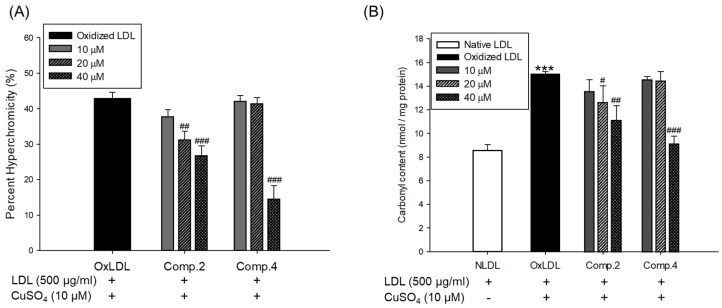
Effect of low concentration of compounds **2** and **4** from *V. rotundifolia* fruit extract on UV absorption and carbonyl content of oxidized LDL. (**A**) Percent of hyperchromicity (absorbance at 280 nm) of oxLDL, and LDL treated with compounds **2** and **4** (final concentration, 10, 20, and 40 μM). (**B**) Carbonyl content determination of native LDL, oxLDL, and LDL treated with compounds **2** and **4** (final concentration, 10, 20, and 40 μM). These data are expressed as mean ± SD of three independent experiments. ***p < 0.001 versus NLDL (native LDL); ^#^*p* *<* 0.05; ^##^*p* < 0.01; ^###^*p* < 0.001 versus OxLDL (oxidized LDL).

**Figure 5:**
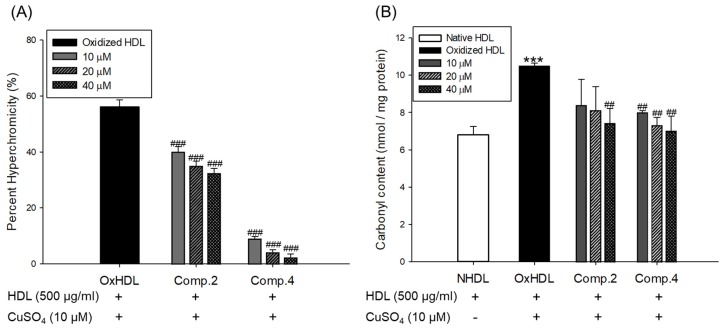
Effect of low concentration of compounds **2** and **4** from *V. rotundifolia* fruit extract on UV absorption and carbonyl content of oxidized HDL. (**A**) Percent of hyperchromicity (absorbance at 280 nm) of OxHDL and HDL treated with compounds **2** and **4** (final concentration, 10, 20, and 40 μM). (**B**) Carbonyl content determination of native HDL, OxHDL, and HDL treated with compounds **2** and **4** (final concentration, 10, 20, and 40 μM). These data are expressed as mean ± SD of three independent experiments. ***p < 0.001 versus NHDL (native HDL); ^##^*p* < 0.01; ^###^*p* < 0.001 versus OxHDL (oxidized HDL).

Oxidized LDL and HDL by copper ion significantly increased hyperchromicity compared with native state (Figure 4A and Figure 5A). However, LDL treated with compound **2** (at 20 and 40 μM) and compound **4** (at 40 μM) and HDL treated with compounds **2** or **4** (at 10, 20, and 40 μM) significantly reduced hyperchromicity compared with the native LDL and HDL (Figure 4A and Figure 5A). In addition, carbonyl content of native and oxidized LDL and HDL was determined by reaction of DNPH with protein-bound carbonyl groups. Carbonyl content of oxidized LDL and HDL was shown to be remarkably higher than that of native LDL and HDL, respectively (Figure 4B and Figure 5B). In contrast, carbonyl content was remarkably reduced when the LDL was present with compounds **2** (at 20 and 40 μM) and **4** (at 40 μM) compared to oxidized LDL (Figure 4B). Treatment of compounds **2** (at 40 μM) and **4** (at 10, 20, and 40 μM) showed a significant recovery of carbonyl content on oxidized HDL by copper ion (Figure 5B).

### 3.3. Inhibitory Effect of Change of Charge by V. rotundifolia Fruit Extract and Its Compounds on LDL and HDL Oxidation 

**Figure 6:**
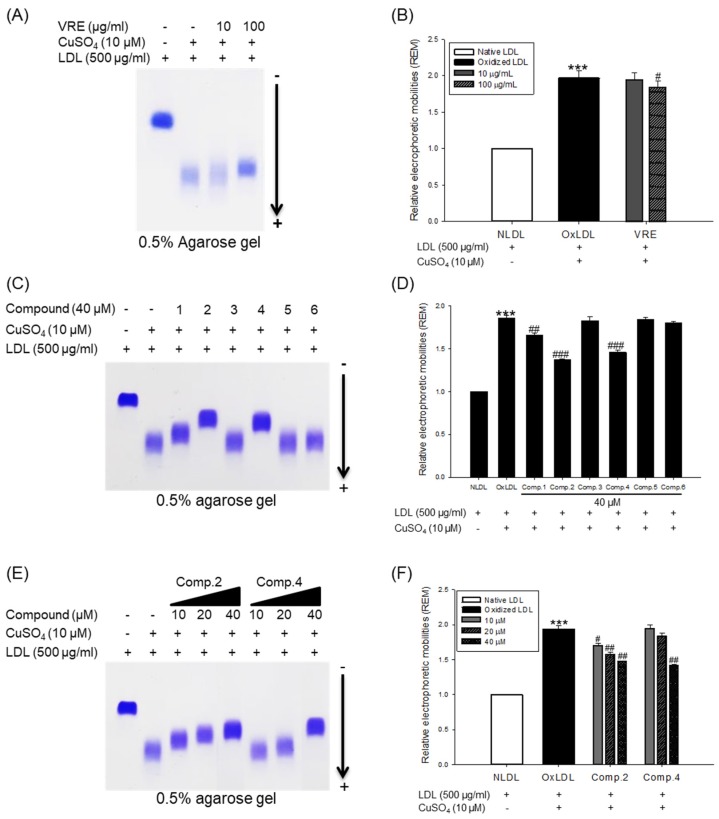
Agarose gel electrophoresis analysis of *V. rotundifolia* fruit extract and its six compounds on Cu^2+^-induced LDL oxidation. (**A**) Electrophoretic mobility profile of LDL, which was treated with *V. rotundifolia* fruit extract (at 10 and 100 μg/mL) in the presence 10 μM copper ion. (**B**) Graph shows the calculation of relative electrophoretic mobility, which was calculated as the distance traveled from the origin. (**C**) Electrophoretic mobility profile of LDL, which was treated with the presence of six compounds (at 40 μM) in the presence 10 μM copper ion. (**D**) Graph shows the calculation of relative electrophoretic mobility, which was calculated as the distance traveled from the origin. (**E**) Electrophoretic mobility profile of LDL, which was treated with the presence of low concentration compounds **2** and **4** (10, 20, and 40 μM) in the presence 10 μM copper ion. (**F**) Graph shows the calculation of relative electrophoretic mobility, which was calculated as the distance traveled from the origin. ***p < 0.001 vs. NLDL (native LDL); ^#^*p* < 0.05 vs. OxLDL (oxidized LDL); ^##^*p* < 0.01 vs. OxLDL; ^###^*p* < 0.001 vs. OxLDL.

**Figure 7:**
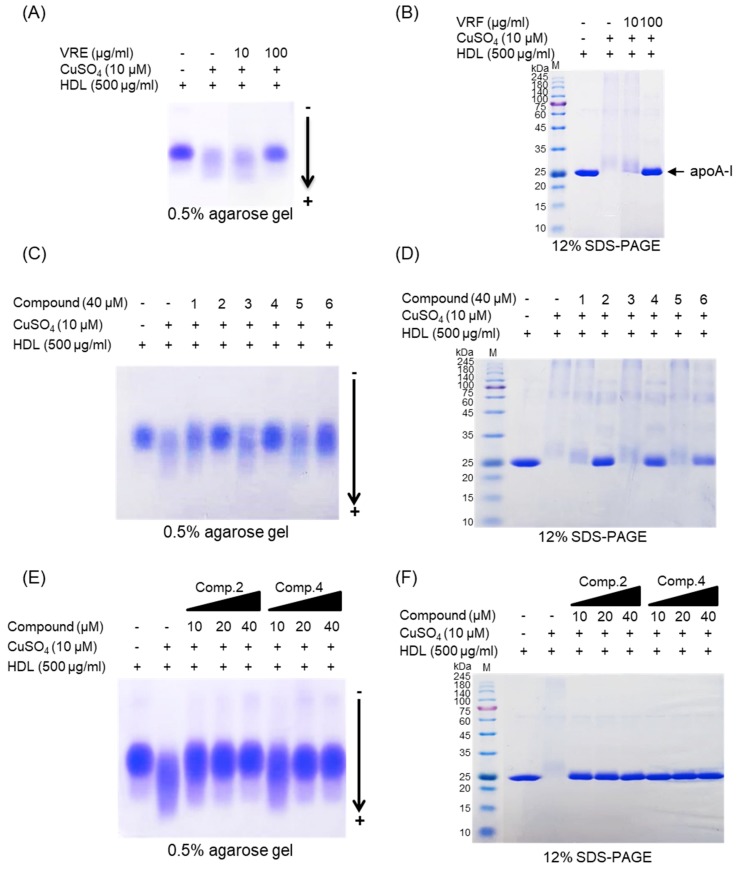
Agarose gel electrophoresis and SDS-PAGE analysis of *V. rotundifolia* fruit extract and its six compounds on Cu2+-induced HDL oxidation. (**A**) Electrophoretic mobility profile of HDL, which was treated with *V. rotundifolia* fruit extract (at 10 and 100 μg/mL) in the presence 10 μM copper ion. (**B**) SDS-PAGE of HDL modified by 10 μM CuSO_4_ for 4 h in the absence or presence of *V. rotundifolia* fruit extract (at 10 and 100 μg/mL). (**C**) Electrophoretic mobility profile of HDL, which was treated with the presence of six compounds (at 40 μM) in the presence 10 μM copper ion. (**D**) SDS-PAGE of HDL modified by 10 μM CuSO_4_ for 4 h in the absence or presence of six compounds (at 40 μM). (**E**) Electrophoretic mobility profile of HDL, which was treated with the presence of low concentration compounds **2** and **4** (10, 20, and 40 μM) in the presence 10 μM copper ion. (**F**) SDS-PAGE of HDL modified by 10 μM CuSO_4_ for 4 h in the absence or presence of low concentration compounds **2** and **4** (10, 20, and 40 μM).

The change of electrical charge on LDL was assessed by agarose gel electrophoresis analysis. The relative electrophoretic mobility (REM) of oxidized LDL was increased almost twofold compared with native LDL (Figure 6A,B). However, when oxidation of native LDL was performed in the presence of *V. rotundifolia* fruit extract (at 100 μg/mL), compounds **2**, **4**, and **6** (at 40 μM), and low concentration of compounds **2** and **4** (at 10 and 20 μM), the REMs significantly reduced compared to the oxidized LDL (Figure 6A−F). The band of OxHDL was more negatively charged than that seen in the case of native HDL, whereas the presence of *V. rotundifolia* fruit extract (at 100 μg/mL), compounds **2**, **4**, and **6** (at 40 μM), and low concentration of compounds **2** and **4** (at 10 and 20 μM) recovered the reduction of positive charge on oxidized HDL by copper ion (Figure 7A,C,E).

### 3.4. Inhibitory Effect of apoA-I Aggregation by V. rotundifolia Fruit Extract and Its Compounds on HDL Oxidation

The inhibitory effects of *V. rotundifolia* fruit extract and six compounds on apolipoprotein A-I (apoA-I) of HDL aggregation were analyzed using SDS-PAGE (sodium dodecyl sulfate-polyacrylamide gel electrophoresis). The multimeric pattern of apoA-I showed in oxidized HDL, which revealed that copper ions promote the aggregation of apoA-I (Figure 7B). However, HDL treated with 100 μg/mL of *V. rotundifolia* fruit extract and 40 μM of compounds **2**, **4**, and **6** inhibited apoA-I aggregation (Figure 7B,D). In particular, the apoA-I band of HDL treated with the 100 μg/mL of extract and 40 μM of compounds **2** and **4** exhibited a similar pattern to that of native HDL (Figure 7D). Besides, the compounds **2** and **4** (at 10 and 20 μM, respectively) showed similar activity (Figure 7F) to that of HDL treated with compounds **2** and **4** (at 40 μM).

### 3.5. Analysis of the Compounds in the Extracts by HPLC-UV

**Figure 8:**
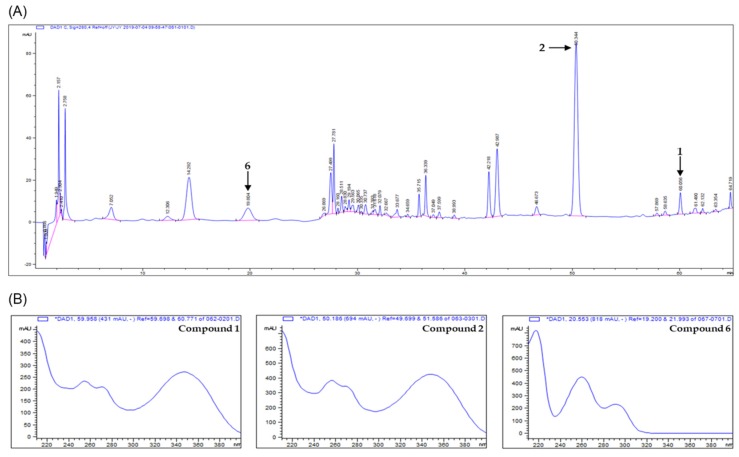
HPLC-UV/PDA chromatogram of the MeOH extract of *V. rotundifolia* fruit. (**A**) HPLC chromatogram of extract of *V. rotundifolia* fruit. (**B**) UV spectra of three compounds.

The analytical method was optimized by adjusting the chromatographic parameters such as solvent, column, gradient range of elution, flow rate, column temperature, mobile phase, and detection wavelength. The acidic water (0.2% formic acid, *v*/*v*) and acetonitrile were used as mobile phase and stationary phase used the C18 column. The various types of compounds were scanned by detecting analytes using PDA detectors in the wavelength range from 190 to 400 nm. The wavelength of 280 nm was chosen as the suitable detection because the resolution and baseline were much better than with other wavelengths in the chromatogram. In the chromatogram, the best optimized method was constructed for proper separation. Analysis of chromatogram was assessed by the ascending, apex, descending regions, and symmetry of peaks. The results of HPLC analysis with *V. rotundifolia* fruit extract showed that they had more than six compounds (Figure 8). Each peak of the chromatogram on the extract was established by spiking and mass spectrometry (MS). In the HPLC chromatogram, three peaks (at 60.056, 50.344, and 19.804 min) were confirmed to correspond to artemetin (**1**) casticin (**2**), and vanillic acid (**6**), respectively. For quantitative analysis of the compounds in the extract, the calibration curves for the three compounds were obtained by plotting the peak area versus the concentration for each analyte by least-square regression analysis. Each calibration equation was obtained using seven levels of concentrations ranging from 0.0078 to 0.5 mg/mL for each analyte. The range of all calibration curves was adequate for the simultaneous determination analysis of three compounds in the sample extract. The linear correlation coefficient (*r*^2^) of calibration curves was higher than 0.99, indicating a good linearity, and the contents of compounds **1**, **2**, and **6** measured based on calibration curve were found to be 1.8375 ± 0.0069, 8.6287 ± 0.0778, and 1.7168 ± 0.0060 mg/g, respectively (Table 1).

## 4. Discussion

*V. rotundifolia* fruit has been traditionally used as a folk medicine to improve premenstrual syndrome (PMS) and cardiovascular disease [44,45]. Casticin, a flavonoid which has been reported to have antioxidant, anticancer, and anti-inflammatory activities, is a main pharmacologically active constituent in the fruit of *V. rotundifolia* [34,46]. However, it remains unclear whether *V. rotundifolia* fruit extract and its constituents have cardioprotective activities through inhibition of oxidation of LDL and HDL or not. In the current study, the inhibitory effects of *V. rotundifolia* fruit extract and its constituents on the oxidation of the human plasma LDL and HDL were evaluated.

Copper sulfate (CuSO_4_) was used to induce LDL and HDL oxidation. In many previous studies, copper has been widely used to initiate LDL and HDL oxidation because a high level of copper ion in serum was reported to be associated with accelerated progression of atherosclerosis [47]. Measurement of the generated conjugated dienes (CDs) and malondialdehyde (MDA) is the most widely used method to measure lipid peroxidation in vitro in spite of the presence of various methods [48]. CDs, which are generated in the early stage of peroxidation of unsaturated fatty acids (PUFAs) in LDL or HDL, have been used to analyze the extent of lipid oxidation. It is produced by rearrangement of double bonds in PUFAs, which could be quantitatively evaluated by measuring the absorbance at 234 nm using UV spectroscopy. The formation of CDs and its lag time are well-known indicators of lipoprotein oxidation and highly associated with the risk of coronary heart disease [49]. Lag time, a reliable marker of LDL oxidation, indicates resistance of LDL to oxidation [37]. Cleavage of the CDs is known to make malondialdehyde (MDA), the end product of lipid peroxidation and a biomarker of oxidative stress. Previous studies reported that an increase in serum MDA level is associated with development of atherosclerosis [50]. In our results, *V. rotundifolia* fruit extract and its compounds remarkably decreased CD generation and reduced MDA formation on oxidized LDL and HDL induced by copper ion (Figure 2A−D and Figure 3A–D). Even low concentrations of compounds **2** and **4** powerfully restricted the generation of CD and MDA (Figure 2E,F and Figure 3E,F). These results indicated that *V. rotundifolia* fruit extract and compounds **2** and **4** could suppress lipid peroxidation through inhibiting CD and MDA formation on copper-mediated oxidized LDL and HDL.

The increase in hyperchromicity reflects exposure to chromophoric aromatic residues through fragmentation and unfolding of the protein by oxidation [51]. Previous studies have reported that the hyperchromicity of oxidized hemoglobin and LDL elevated compared with the native state [52,53]. In the present results, UV spectra of oxidized LDL or HDL increased hyperchromicity by up to 53% and 56%, respectively (Figure 4A and Figure 5A), which is in good agreement with the previously published experimental results. However, the oxidized LDL and HDL treated with compounds **2** and **4** (at 10, 20, and 40 μM) significantly reduced hyperchromicity (Figure 4A and Figure 5A). These findings showed that compounds **2** and **4** prevented modification including unfolding and fragmentation of oxidized LDL and HDL by copper ion.

The oxidation of proteins was known to result in the production of carbonyl groups, which could be used as a relative marker of oxidative stress injury [46]. Previous studies have demonstrated that carbonyl content increased in oxidized hemoglobin, LDL, serum, and plasma of patients with type 2 diabetes [52,53,54,55]. Our current results showed that the level of carbonyl content of oxidized LDL and HDL was remarkably higher than that of native state (Figure 4B and Figure 5B). On the other hand, carbonyl content was remarkably decreased when LDL was treated with compounds **2** (at 20 and 40 μM) and **4** (at 40 μM) and HDL was treated with compounds **2** (at 40 μM) and **4** (at 10, 20, and 40 μM). Our results demonstrated that compounds **2** and **4** were good scavengers of carbonyl in oxidized LDL and HDL.

When LDL is oxidized, the positive charge of ε-amino groups in lysine residues of apoB-100 is known to be neutralized [56]. Several studies demonstrated that the oxidized LDL had a higher negative charge than native LDL [57]. Accordingly, REM (relative electrophoretic mobility) is known to increase on oxidation of LDL. In our study, *V. rotundifolia* fruit extract and compounds (**1**, **2**, and **4,** at 40 μM) remarkably recovered REM of oxidized LDL (Figure 6A–D). Surprisingly, compound **2** showed strong activities even at 10 and 20 μM (Figure 6E,F), which agreed with the results obtained by CD, TBARS, hyperchromicity, and carbonyl content assay for its inhibitory effects on LDL oxidation.

Oxidized HDL is known to increase denaturation of apoA-I (apolipoprotein A-I), along with increasing the negative charge and lipid peroxides in comparison to native HDL [58]. ApoA-I, the main protein component of HDLs, plays a key role in mediating several beneficial effects in HDL including anti-inflammatory, antithrombotic, and antioxidative activities. Modification of apoA-I is directly associated with formation of dysfunctional HDL, which has proinflammatory and proatherosclerotic properties. In our agarose gel electrophoresis analysis, the band pattern of oxidized HDL diffused more than the native HDL. It indicated that negative charges on the oxidized HDL increased (Figure 7A), suggesting increase of multimeric apoA-I (Figure 7B). However, HDL treated with 100 μg/mL of *V. rotundifolia* fruit extract and 40 μM of compounds recovered the change of charge and suppressed apoA-I aggregation in a similar way to that of native HDL (Figure 7A–D). In particular, compounds **2** and **4** showed the most potent activities even at low concentrations (Figure 7E,F). These results suggested that *V. rotundifolia* fruit extract and compounds **2** and **4** remarkably can aid to prevent dysfunctional HDL formation. Electronegative LDL is known to have a lower binding affinity for LDL receptors than normal LDL and delays residence time in the blood circulation, which promotes further modification of electronegative LDL, increasing the risk for inflammation and atherosclerosis [59]. It has been reported that electronegative HDL shows a decrease of antioxidant activity, antiapoptotic activity, cholesterol efflux capability, and anti-LDL oxidation. These results are associated with an increased risk of coronary artery disease [60].

To investigate the amounts of the active compounds in the extract of *V. rotundifolia*, a simple, rapid, and reliable analytic method using HPLC-DAD was developed. In the developed method, three compounds from *V. rotundifolia* fruit, artemetin (**1**) casticin (**2**), and vanillic acid (**6**), were detected (Figure 8). The method was successful for the quantitative determination of the major compounds in the sample extract (Table 1). In this study, casticin was found to be a major one in the extracts. Casticin is also known as a major component in *V. agnus-castus*. However, the content of casticin in *V. agnus-castus* had a range of 6–14.9 mg/100g of dried samples whereas that in *V. rotundifolia* analyzed in this study was 43 mg/100 g of dried sample, suggesting its advantage in terms of the contents of active compounds [61,62].

## 5. Conclusions

To the best of our knowledge, this is the first study on inhibitory effects of *V. rotundifolia* fruit and its constituents including five flavonoids and one phenolic acid on LDL and HDL oxidation. Our findings suggested that *V. rotundifolia* fruit extract and compounds **2** (casticin) and **4** (luteolin) had strong activities toward LDL and HDL oxidation, and might have potential as good supplements for reduction of risk for atherosclerosis. Casticin was found to be present in relatively large amounts in the extract while luteolin could not be detected under the developed condition due to the limited amount in the extracts. Further studies dealing with other possible antiatherosclerosis-related mechanisms and in vivo efficacies for compounds **2** and **4** will be required.

## Figures and Tables

**Table 1:** Calibration curves and contents of the compounds in *V. rotundifolia* fruit.

Analytes	Calibration Curve	*r* ^2^	Content (mg/g)
Artemetin (**1**)	y = 1,539,769.3330x − 142.5713	0.9994	1.838 ± 0.007
Casticin (**2**)	y = 2,611,350.7177x − 145.8017	0.9997	8.629 ± 0.078
Vanillic acid (**6**)	y = 2,445,942.6241x − 163.7577	0.9993	1.717 ± 0.006
